# Primary health care accessibility challenges in remote indigenous communities in Canada's North

**DOI:** 10.3402/ijch.v74.29576

**Published:** 2015-10-26

**Authors:** Tim Michiel Oosterveer, T. Kue Young

**Affiliations:** 1 MSc Program in Health Sciences (International Public Health), Vrije Universiteit, Amsterdam, the Netherlands; 2 School of Public Health, University of Alberta, Edmonton, AB, Canada

**Keywords:** Northwest Territories, Canada, primary health care, accessibility, remote

## Abstract

**Background:**

Despite many improvements, health disparities between indigenous and non-indigenous populations in Canada's North persist. While a strong primary health care (PHC) system improves the health of a population, the majority of indigenous communities are very remote, and their access to PHC services is likely reduced. Understanding the challenges in accessing PHC services in these communities is necessary to improve the health of the population.

**Objective:**

The objective of the study was to document and analyze the challenges in accessing PHC services by indigenous people in remote communities in Canada's Northwest Territories (NWT) from the perspectives of users and providers of PHC services.

**Methods:**

Using explorative, qualitative methods, our study involved 14 semi-structured interviews with PHC service providers (SPs) and service users (SUs) in 5 communities across the NWT which varied according to population, remoteness, ethnic composition and health care resources. The interview guide was developed after key informant consultations.

**Results:**

Both SPs and SUs understood the constraints in providing equitable access to PHC services in remote communities. The provision of emergency care was found to be particularly challenging, because of the lack of qualified staff in the community and the dependence on aeromedical evacuations. Wider dissemination of first aid skills among community members was seen to cover some gaps and also increase self-confidence. For non-emergency care, the need to travel outside the community was generally disliked. All recognized the need for more preventive services which were often postponed or delayed because of the overwhelming demand for acute care. As long as services were provided in a community, the satisfaction was high among SUs. SPs appreciated the orientation they received and the ability to build rapport with the community.

**Conclusions:**

Northern SUs and SPs generally acknowledge the health consequences of living in remote communities. The generally high level of satisfaction attests to the overall effectiveness of PHC in the region despite geographical remoteness. Many improvements could be made in terms of upgrading the clinical skills of SPs and community members, improving staff retention, extending the frequency and duration of community visits and providing more attention to preventive services.

Equitable access to primary health care (PHC) services is essential to reducing health disparities between indigenous and non-indigenous populations in the circumpolar region. Understanding the underlying factors contributing to PHC access is the first step towards a more equitable distribution of health within and across populations (1,2). The circumpolar regions are vast and host to a variety of indigenous populations. Many indigenous communities are remote, generally lack adequate infrastructure and endure chronic shortages of health care personnel (3,4). Despite having the highest per capita health expenditures in Canada, the North reports poorer health outcomes than the rest of Canada, especially among its indigenous people (5,6). The transition from a traditional to a more modern way of life has resulted in an increasing burden of chronic diseases (3,7), increasing further the pressure on the already limited available resources. Tudor Hart's (8) “inverse care law” states that “the availability of good medical care tends to vary inversely with the need for it in the population served”. Evidence suggests that the “inverse care law” may well apply in Canada's Arctic.

As stated in the 1978 Alma Ata Declaration, PHC is the key strategy to ensure “health for all” (9). PHC is essential care that is universally accessible, it is a community member's first contact with the health system and it brings health care closer to home. A strong PHC system is best suited to increase the health of the indigenous populations in northern Canada in a sustainable manner. Increased accessibility to PHC in Canada's North could reduce major causes of death, lower costly medical evacuations and reduce health disparities between populations (1,2).

For the purpose of this study, we adopted the definition of accessibility used in the United States Institute of Medicine's report on access to health care – “the timely use of personal health services to achieve the best possible outcomes” (10). The PHC system in northern Canada is fundamentally different from that in existence in much of urban, southern Canada (5,6). It is nurse based, with nurses stationed in health centres performing many tasks that are traditionally the physicians’ preserve. This is a form of “task-shifting” that is common in many underserved regions globally, whereby specific tasks are moved from highly qualified health professionals to health workers with shorter training in order to make more efficient use of the available human resources (11). There is yet another category of front-line health workers – called community health workers (CHWs) – who not only provide first contact care in those small communities without resident nurses but also help in bridging cultural barriers between the health system and users (12).

The objective of the study was to document and analyze the challenges in accessing PHC services by indigenous people in remote communities in Canada's Northwest Territories (NWT) from the perspectives of both service users (SUs) and service providers (SPs). Such an understanding is critical to the development of effective intervention strategies to increase accessibility to PHC services. The study was conducted by the first author (TMO) as part of the requirement for his master's degree in global health.

## Methods

This explorative qualitative research project using the grounded theory approach (13) comprised literature review, key informant consultations, participant observations and 14 semi-structured interviews with PHC SPs and SUs in 5 communities in the NWT of Canada conducted between September and December 2014. TMO travelled extensively across the territory prior to the interviews to gain insight into the regional diversity within NWT regarding indigenous cultures and health care delivery.

**Fig. 1:**
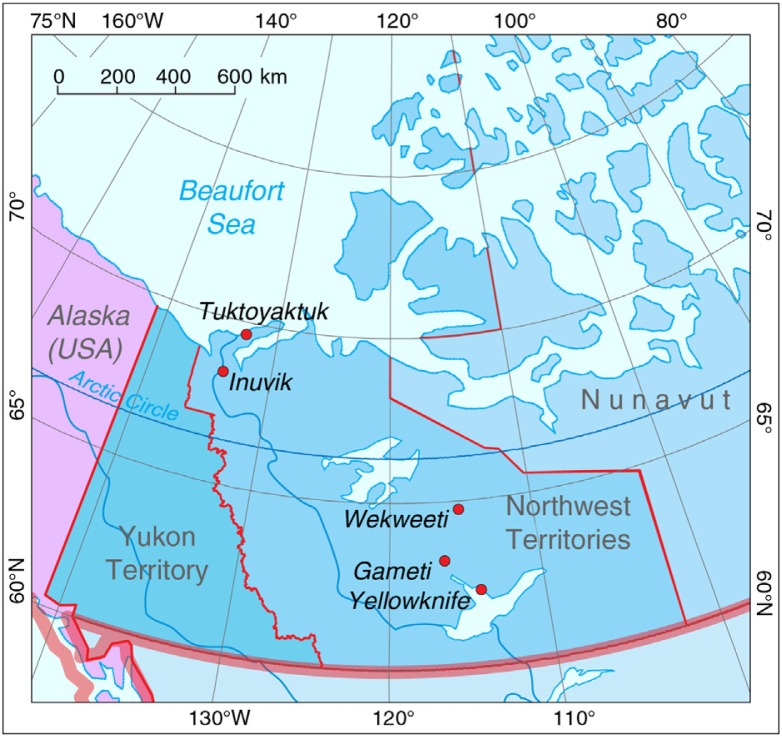
Map of Northwest Territories showing location of the 5 study communities.

From preliminary community visits and consultations with key informants, 5 communities were selected on the basis of population size, geographic remoteness, mix of indigenous and non-indigenous ethnic groups and health care resources (Fig. 1). The 5 communities were categorized into 4 levels, based on the health care resources available: 1 part-time nurse present at the health centre (level 1), 1 full-time nurse present at a health centre (level 2), multiple full-time nurses present at a health centre (level 3) and a hospital staffed by physicians (level 4) (Table I). The key informants were drawn from decision-makers and providers in the territorial government and regional health authorities who were long-term residents of the NWT. They also suggested names in the 5 communities as potential interviewees. On arrival at the community, these individuals were contacted and their consent to participate obtained.

**Table I:** Characteristics of selected communities participating in the study

Level	Community	Location	Population	Health care resources	Road access
1	Wekweèti	190 km north of Yellowknife	140 (~100% Dene)	Health clinic with 2 rooms at local government office; CHW on site with periodic visits by nurse and GP	Ice road in winter; otherwise by air only
2	Gamèti	230 km north of Yellowknife	250 (~90% Dene)	Health centre with 1 full-time nurse	Ice road in winter; otherwise by air only
3	Tuktoyaktuk	1,140 km north of Yellowknife; 130 km north of Inuvik	850 (~90% Inuit)	Health centre with 5 nurse positions, usually only 2–3 nurses present	All weather road under construction; otherwise by air only
4	Inuvik	1,090 km north of Yellowknife	3,460 (40% Inuit, 20% Dene, 5% Métis)	Inuvik Regional Hospital (50 beds) with visiting specialist services; primary care clinic in town also serving outlying communities	Road-accessible year round
4	Yellowknife	1,500 km by road to Edmonton, 990 km by air	19,230 (15% Dene, 5% Inuit, 5% Métis)	Stanton Territorial Hospital (90 beds) with some specialist services; primary care clinics in city also serving outlying communities	All weather road to Edmonton

CHW, community health worker; GP, general practitioner.

Population data (rounded) are from the 2011 Canada Census (www12.statcan.gc.ca/census-recensement/2011/dp-pd/prof/index.cfm?Lang=E).

The proportions of Aboriginal identity groups (rounded) are from the 2011 National Population Survey community profiles (www12.statcan.gc.ca/nhs-enm/2011/dp-pd/prof/index.cfm?Lang=E).

Inuit in Inuvik and Tuktoyaktuk are predominantly Inuvialuit. Dene in Wekweèti and Gamèti are Tlicho.

Interview participants included SUs who had been living in the NWT and used PHC services in the past 5 years. SUs were offered a Can$50 coupon from a local store as an incentive. SPs comprised general practitioners, registered nurses and nurse practitioners employed in community health centres and CHWs.

All participants signed a written informed consent. Eleven of the 14 participants were female (79%) with a mean age of 49.4 years, while the 3 male participants had a mean age of 54.3 years. Eight of the 14 interviewees were SUs, while 6 were SPs. For confidentiality reasons, the participants were not further characterized beyond their occupational status in this paper.

The 14 semi-structured interviews were conducted by TMO. They were supplemented with fieldnotes of meetings with health service experts, government officials and community members. Separate interview guides were developed for SUs and SPs, focussing on their respective experience with the clinical encounter and the factors that affected that experience. Each community visit lasted between 3 and 8 days. All data were coded and analyzed by TMO using the online qualitative and mixed methods analysis software Dedoose (www.dedoose.com).

After each interview, the recording was digitally transcribed verbatim by the interviewer and key points were collected on a post-interview comment sheet. An iterative process of concurrent data collection and data analysis was maintained. The transcriptions were repeatedly analyzed with coding. This information was then used to specify the questions, concepts and topics for the next interview. The interviews continued until the desired number of participants per stakeholder group had been reached.

The data analysis aimed to search for and explain patterns in the data. The sensitizing concepts from the literature review and key informant consultations formed the conceptual model, and this model was used during the conduct and analysis of the first semi-structured interviews. During this explorative phase, the analysis was inductive with “open coding” to create and adjust labels and codes. These codes were collected in a code file. In the following specification phase, there was hierarchical axial coding, using the labels and codes collected, to identify groups and topics. During the following phase, the coding became more selective to include all relevant data according to the identified topics and the last phase was predominantly deductive to test the findings that were generated.

Ethical approvals were granted by the academic institutions of the graduate student (TMO) and the principal investigator of the Canadian Institutes of Health Research team grant which funded the study (TKY). The study also underwent review by the ethics review board of the Stanton Territorial Health Authority. A research license was granted by the Aurora Research Institute, the licensing agency under the NWT *Scientists Act*, which instituted its own consultation process with the participating communities.

## Results

Both SPs and SUs understood that the provision of completely equal and equitable access to PHC services across the NWT was unrealistic due to the geographical characteristics and small population size of the remote communities. Still, a need was expressed by both groups to strive for timely, risk- and barrier-free, and appropriate PHC as close to home as possible.

### Access to emergency care

Access to health services in emergency situations is challenging and generally considered poor in the small and remote communities. SUs and SPs described the situation using terms such as “scary,” “far from ideal” or “unacceptable.” The first challenge described was a lack of qualified health staff when they were needed in emergency situations. This was worsened through inefficient scheduling of mobile clinics that overlapped in the level 1 community. Every 6 weeks, a nurse came in for 2 weeks and a physician for 3 days. When they overlapped, it resulted in cramped working space. Without overlap, the total number of SP days in a community would be increased. The second challenge was the high utilization rate of medical air travel. Communities in levels 1–3 mostly fly in except for a few mid-winter weeks when there is an ice road. Because of adverse weather conditions, it may take anywhere between 4 hours and 6 days before a plane can arrive in an emergency. The third challenge reported was the lack of community members with training in first aid. This was particularly important for the level 1 community. When there were no visiting SPs, the wider dissemination of first aid skills among community members would fill in some of the health care gaps.

So I call for a plane, let the emergency room in Yellowknife know that the patient is coming, and the plane comes and picks them up. But if you had a heart attack here, you would never survive. – SP [nurse]

### Delivery of non-urgent care

A different set of challenges was reported relating to health care in non-emergency situations. Common to communities in all levels were issues related to the lack of confidentiality. This was particularly true in the smaller communities without resident nurses and served by a CHW from the community. Several interviewees reported not feeling safe in sharing their health issues with people at the health centre as they feared that these would then be shared with other community members, which might lead to social exclusion. SUs reported avoiding CHWs whom they did not trust and would rather wait for nurses from outside the community to arrive, which could be weeks away.

… sometimes the families, they get together and they talk about what's going on in the community. And I heard that they talk about some health, some people's health problems. So that's why I prefer the nurse or the doctor. – SU

This leads to the second challenge – the difficulties experienced by indigenous SPs to work in their own communities. An indigenous SP returning to his/her tight-knit community might face extra social pressure from the SP's family and friends in the community who had high expectations.

A Dene nurse may have a little more problems in the beginning, because of trust …. ‘Oh she is going to tell her friends about us or people that we know’. – SP [nurse]

On the contrary, familiarity with community dynamics could be an advantage that an indigenous SP had over a visiting non-indigenous SP from outside.

Other challenges reported were similar to those related to emergency health care: low retention of health professionals in the community and a high rate of use of expensive medical air travel for non-emergency situations. Understaffing, low retention and a high turnover rate of SPs is a chronic persistent problem throughout the NWT. The need for advanced clinical training in locations far away from specialists and hospitals is one reason why it is difficult to find and retain SPs in level 1–3 communities. This is aggravated by a perceived lack of support by the regional centres. Almost all SPs working in indigenous communities are non-indigenous, with added cultural and linguistic barriers. On the contrary, some SUs prefer this as it creates a more “confidential” situation. Physicians working in regional hospitals providing clinical support to nurses and CHWs in the communities do not always trust the judgement of other providers:

There could probably be fewer medevacs because when I medevac it would be a patient that I feel needs to be seen by somebody. If I was there I would see the patient myself. – SP [GP]

### Preventive health programmes

The lack of preventive health programmes in the communities was widely recognized by the SPs, which they attributed to the high workload in providing acute care. Almost all their time is spent in running “sick clinics”, with preventive services provided only when there is extra time available, which is rare. In addition, there are few public meetings in the communities that focus on wellness issues, an opportunity to provide public education and promote prevention. The need for public health is highlighted by the rapid changes in lifestyles and the accompanying modern lifestyle-related diseases. Although the majority of SUs and SPs recognized the increasing importance of modern lifestyle-related diseases in their community, they did not have a frame of reference to compare the present situation with that in the past, or between indigenous and non-indigenous people. The 2 most commonly mentioned unhealthy modern lifestyle mentioned by the SUs were reduced household and leisure-time physical activity and an increase in consumption of frozen/canned/processed food from a store.

Because I think the cultural food is healthier then the processed. Because they use lots of chemicals and stuff. What the elders believe, like, ever since there's processed meats and stuff, people are getting more sicker. More cancer and stuff like that. – SU

The majority of SPs supported this finding and explained that there was very little time to educate community members about healthy lifestyles, because of the workload and the priority of acute care.

With one week every six weeks, the public health programs suffer. […] According to Canadian standards, to keep the community up to the Canadian standards, they need more health time and a health professional here more. So a lot of the public health, except for the communicable disease and immunizations, is behind. – SP [nurse]

Another challenge was communication. Many elders speak the local language while many children speak English. As the elders used to teach children about health and the “old ways” of living, they felt that they had lost this ability and described a loss of culture because of it.

It's kind of hard to talk to them in our language because most of them speak English. So it's not good for elders to teach kids like that. They can look and that's all, they don't understand. They won't understand them. – SU.

Barriers in communication also existed between some indigenous community members and English-speaking staff at hospitals and health centres. While translators and escorts were often provided, the community's satisfaction with these services varied. There were also communication barriers even among community members. Elders used to play a pivotal role in engaging with community members, but many interviewees said they had become too old or too few to take on that role. Some had described a tendency towards a more “private” and “own household-oriented” culture within the community. Multiple interviewees expressed their concerns about this lack of communication among community members, which did not help with the increasing burden of mental health problems that many people were struggling with.

They don't talk about themselves and too many people are killing themselves. – SU

### Positive experiences

The preparation and education an SP received before working in the NWT was generally considered a positive experience. SPs stressed the importance of good preparation because task-shifting within the health care system had broadened their scope of practice extensively in the isolated communities. However, to benefit the most from education, preparation courses, orientation and support services, one needed to possess the right personal skills and attitude.

Oh my god yes, definitely helpful and necessary. Because here I am the only person. So if somebody has a heart attack or there is a motor vehicle accident with two people dead and three people alive and they are all mangled up. And I have to deal with that. – SP [nurse]

The second positive experience reported by some SPs was the satisfaction from being able to build rapport with SUs, which required the SPs to engage and invest in the relationship. This proactive strategy was described as essential for SPs who came from outside the community. When trust had been established, many interviewees reported that there was less fear for potential breaches of confidentiality.

And going to the community, where people, you know, are not sure what they are going to do. Get to know who runs the community, who are the leaders in the community that can make it or break it for you. – SP [nurse]

SUs were overall satisfied with those services provided in the community. The priority for SUs was to be able to get health services without leaving the community.

You know, we shouldn't have to move or be sent out of our community just to get decent medical attention. – SU

## Discussion

This explorative interview-based qualitative study identifies several challenges in accessing PHC services in remote indigenous communities of the NWT reported by both SUs and SPs. These challenges have been found in emergency and non-emergency situations, and also in public health. As a small exploratory study with time constraints, not all relevant or important viewpoints can be brought to light. However, the broad issues identified can be followed up by more in-depth studies using additional methods such as ethnography. As part of the larger programme of research, a providers’ survey in the NWT had been conducted early in 2015 which addressed more specifically issues related to medical evacuations, inter-professional communication and clinical support between regional centres and the community health centres.

The lack of resources in the smaller indigenous communities associated with a greater need for accessible primary care services confirm Hart's Inverse Care Law (8). Increasing resources for indigenous people living in the remote North should therefore be a high-priority health care policy.

The majority of interviewees had a good understanding of the causes of poor health in the North, such as widespread poverty, high unemployment, low level of education, limited resources in the communities and high rates of alcohol and drug abuse. From our own observations, these problems are not unique to the North but appear to be exacerbated by the extreme environmental conditions such as the severe cold and extended darkness in the winter, and the destabilizing effects of rapid social and cultural change.

The majority of community members appreciated the fact that equitable and equal access to PHC services is not a realistic goal given the geographic characteristics of some communities. However, there is a definite gap between community expectations and the actual delivery of health services. The accessibility to emergency care in the smallest community has been described as seriously problematic because of the part-time presence of nursing staff. Continual accessible care could only be partially provided by a CHW. Furthermore, a CHW does not necessarily increase accessibility due to confidentiality issues and irregular absence. The benefits of using CHWs in providing PHC services in small communities without full-time presence of nursing staff are well established (12), but issues of confidentiality, training needs and clinical support from a distance must be addressed. Community members can all benefit by more widespread dissemination of emergency response skills (14). This would increase self-sufficiency and reduce the sense of insecurity within the communities. Such first aid skills, however, cannot replace more advanced knowledge and skills that only a nurse or physician could provide.

Within the non-emergency domain, a major challenge was the regular, time-consuming and extended medical travels to larger communities for non-emergency care. This separates families, creates a loss of income and requires people to stay in unfamiliar places. Accessibility to non-emergency care, as with the case of emergency care, is compromised by the high turnover rate of SPs.

The impact of the health transition highlights the importance of preventive health programmes. Yet, this is routinely postponed or cancelled because of the high workload and the priority of acute care. In the long term, the burden of chronic diseases and other “new” health issues is expected to further increase. What is not a priority now needs to become a priority if emerging epidemics in the future is to be averted. The communication barriers between the elders who are holders of traditional knowledge and other community members and SPs are well recognized. Because of this language barrier, the elders’ role in educating others about maintaining health and wellbeing has been eroded.

While SUs did not specifically identify cultural competence as a requirement for SPs, SPs themselves generally recognized that their ability to function adequately in the communities would benefit from cultural training and orientation prior to beginning their work. Nevertheless, the single highest priority for both types of stakeholders is increasing and keeping directly accessible services within the community by increasing human resources.

The generally high level of satisfaction with the accessibility of PHC services provided within a community attests to the overall effectiveness of PHC in this region despite the geographical remoteness. This finding is in accordance with community satisfaction surveys conducted by the NWT Department of Health and Social Services (15). Community members, however, do not enjoy receiving care outside the community and the reliance on both emergency and non-emergency medical travel. They have good rapport with the predominantly non-indigenous SPs and they accept ethnic differences because there is a lower risk of confidentiality breach or social complications. There is an expectation that SPs need to be engaged with the community members to build rapport. SPs who feel they are appreciated by the community are more likely to stay longer, contributing to reducing the chronic retention problem. The majority of SPs recognize the importance of good preparation to work and keep working in remote communities. Existing orientation programmes and in-service courses provided by the health authorities appear to be adequate.

While the issue of accessibility to PHC in the North is regularly addressed by a variety of national and regional health surveys, their scope is limited (16). Qualitative data thus play an important role in providing context and a deeper understanding of the perspectives of providers and users. Given the diversity of ethnicities and community types, the 5 communities selected representing 4 levels of health care resources available may not adequately cover the entire health care experience of indigenous people in the NWT.

Based on the issues that emerged from the interviews, improvements to the existing system could be made in terms of upgrading the clinical skills of SPs and community members, improving staff retention, extending the frequency and duration of community visits and providing more attention to preventive services. Finally, health care delivery in the NWT can benefit from comparative health system research in other circumpolar regions (5) and indeed any region globally where the population is widely dispersed in small and remote communities.
